# DOMAS: a data management software framework for advanced light sources

**DOI:** 10.1107/S1600577524000043

**Published:** 2024-02-01

**Authors:** Hao Hu, Lei Lei, Haofan Wang, Bo Zhuang, Ruojin Zhang, Qi Luo, Xiaokang Sun, Fazhi Qi

**Affiliations:** a University of Science and Technology of China, Hefei, Anhui 230026, People’s Republic of China; b Institute of High Energy PhysicsChinese Academy of Sciences, Beijing 100049, People’s Republic of China; c China Spallation Neutron Source Science Center, No. 1 Zhongziyuan Road, Dalang, Dongguan, Guangdong Province 523803, People’s Republic of China; d ShanghaiTech University, Shanghai 201210, People’s Republic of China; e Zhengzhou University, Zhengzhou, Henan, People’s Republic of China; Australian Synchrotron, Australia

**Keywords:** data management, metadata catalogue, data transfer, data service

## Abstract

In recent years, China’s advanced light sources have entered a period of rapid construction and development. As modern X-ray detectors and data acquisition technologies advance, these facilities are expected to generate massive volumes of data annually, presenting significant challenges in data management and utilization. These challenges encompass data storage, metadata handling, data transfer and user data access. In response, the Data Organization Management Access Software (DOMAS) has been designed as a framework to address these issues. DOMAS encapsulates four fundamental modules of data management software, including metadata catalogue, metadata acquisition, data transfer and data service. For light source facilities, building a data management system only requires parameter configuration and minimal code development within DOMAS. This paper firstly discusses the development of advanced light sources in China and the associated demands and challenges in data management, prompting a reconsideration of data management software framework design. It then outlines the architecture of the framework, detailing its components and functions. Lastly, it highlights the application progress and effectiveness of DOMAS when deployed for the High Energy Photon Source (HEPS) and Beijing Synchrotron Radiation Facility (BSRF).

## Abstract 2

DOMAS is a data management software framework specifically designed and developed for advanced light sources in China. It effectively tackles challenges related to data storage, metadata cataloguing, data transfer and data access.

## 1. Introduction

### 1.1. Advanced light sources in China

Advanced light sources, including X-ray free-electron lasers (Bilderback *et al.*, 2020 ▸) and synchrotron radiation light sources based on the diffraction-limited storage ring (Hettel, 2014 ▸), have characteristics such as high brightness, low emittance and high coherence (Huang *et al.*, 2021 ▸). These facilities can meet the experimental methodology requirements of high throughput, multi-modal fusion, ultrafast frequency, *in situ* dynamic loading.

Advanced light sources provide an advanced experimental platform for many basic-science and engineering research fields such as materials science, chemical engineering, energy environment, biomedicine, aerospace, *etc*., and provide strong support for breakthrough innovations in strategic research fields related to economic, social and industrial development.

China is currently experiencing a rapid development phase for advanced light sources. For instance, the Shanghai-XFEL Beamline Project (SBP) and Shanghai Soft X-ray Free Electron Laser facility (SXEFL) (Zhao *et al.*, 2019 ▸) have transitioned into the operational phase. The Shanghai Light Source II (SSRF-II) (Xu & Xiao, 2023 ▸) has initiated trial operations for user experiments. The construction of the High Energy Photon Source (HEPS) (Jiao *et al.*, 2018 ▸; Jiao & Bai, 2022 ▸) and Shanghai High Repetition Rate XFEL and Extreme Light facility (SHINE) (Zhao & Feng, 2018 ▸) is progressing swiftly. Additionally, the Hefei Advanced Light Source (HALF) (Bai & He, 2021 ▸) has officially received state approval to commence the construction phase. Upon the completion and commissioning of these facilities, the data rates and volumes generated are projected to increase by three to four orders of magnitude compared with the first- and second-generation light sources. The projected increase in annual data throughput marks our entry into the ‘Exascale’ era (Dong *et al.*, 2022 ▸).

### 1.2. Challenges and motivations

It is expected that advanced light sources will generate massive data every year, reaching the PB level or even hundreds of PBs. This represents an explosive growth in both data rate and total data volume. Traditional data management methods can no longer meet the demands of managing and processing such large volumes of data (Wang *et al.*, 2018 ▸). Experimental users are incapable of handling data copying manually and preparing the software environment and computing resources required for data analysis. Therefore, there is a need to change the data management model, and provide experimental users with one-stop, automated services covering the scientific data lifecycle, including data acquisition, data storage and management, data download, data processing and analysis (Hu, Qi*et al.*, 2021 ▸). It will significantly improve the efficiency of scientific research carried out on advanced light sources.

To address the challenges of big data in photon science, HEPS has begun to collaborate with facilities such as SHINE, SSRF and HALF to jointly develop scientific software and systems that cover the entire lifecycle of scientific data, including control and data acquisition software (Mamba) (Liu *et al.*, 2022 ▸), data management system, and data analysis integrated software system (Daisy) (Hu, Li *et al.*, 2021 ▸). As the most fundamental and important part, the data management system is involved in all stages of the experiment – before, during and after. It is the basic prerequisite for efficient data access and quick analysis, open sharing and utilization.

In terms of data management, several relevant research and developments are worthy of note: the ICAT framework (https://icatproject.org/), developed at the Science and Technology Facilities Council (STFC) in the UK and implemented at Diamond Light Source, as well as the ISIS neutron facility and the Central Laser Facility at the Harwell complex. The ICAT system aims to catalogue the data through the development of a metadata database, linking all aspects of the research lifecycle from proposal through to data and articles publication, and allowing them to be accessed by users conveniently for their data evaluation. SciCat, developed by PSI (Paul Scherrer Institute), ESS (Europe Spallation Source) and MAX IV, is an open-source (https://github.com/scicatproject) framework with micro-service architecture and latest technologies, and aimed at the management of the whole data lifecycle. The National Synchrotron Light Source II (NSLS-II) has deployed a comprehensive data acquisition and management system (https://nsls-ii.github.io/index.html) at various beamlines.

However, we found that these open-source data management frameworks are not suitable for application and promotion in light sources in China. The CSNS-I, the first phase of the China Spallation Neutron Source (CSNS), adopted ICAT to develop the data management system, but relational databases proved to be inflexible in managing diverse and complex metadata. SSRF developed its own data management system based on SciCat, but it was found that more time and energy on the secondary development is required for adapting and deploying the framework to the facility, requiring data management personnel to be proficient in relevant programming techniques. We recognized that light sources should not repeatedly invest manpower in the development of data management to adapt the framework to their own facilities. We need a more universal and flexible data management software framework that can be adapted to each facility with low manpower cost. Furthermore, these frameworks overlooked the role of the data transfer module in data management and have not included it in the framework.

Through our research, we found that advanced light sources face both common demands and challenges in data management, and also have their own unique characteristics. Therefore, we developed the data management software framework DOMAS, responsible for automating the organization, transfer, storage and distribution of scientific data. DOMAS provides common basic modules and universal interfaces for data management. Only with parameter configuration and minimal code development within DOMAS can a data management system be quickly established for a light source facility or a beamline.

## 2. Overall design

### 2.1. Data management requirement

To track and manage the entire lifecycle of scientific data from acquisition to storage, transmission, analysis and publication for advanced light sources, the main tasks include: establishing standards and specifications related to scientific data management to form the policy basis for data management work; obtaining metadata from other subsystems, including data acquisition system (DAQ), storage, user service, data analysis, *etc*.; designing the metadata catalogue for fast and efficient metadata storing and searching; designing data storage directories on the file system and setting access control; providing standard interfaces to meet the data or metadata access needs of various systems or modules; CD data transfer tools to move data between different storage media; providing data services for experimental users, including data access, download, analysis and tracking.

### 2.2. Design concept

From the perspective of the functional requirements of data management software, the common core modules include: metadata catalogue, data transfer, metadata acquisition and processing, and data portal. We design a software framework DOMAS with aims to design, refactor and encapsulate these four core modules so that each module can be deployed and run independently, with low coupling between modules, using standard interfaces, and configured to meet the different needs of each facility at various stages of data management.

(1) Metadata catalogue – responsible for storing metadata into a database and providing RESTful APIs to access the metadata. Given that each light source facility might have diverse data management requirements, resulting in different schema designs for the metadata database, our framework offers a visualized tool for generating RESTful APIs. This tool enables data management developers to design metadata models and structures via a website, thereby automatically generating RESTful APIs for metadata access.

(2) Data transfer – facilitates the automatic migration of data between different levels of storage media. Due to the huge amount of data produced by advanced light sources, these facilities usually have a hierarchical storage design for reliable data storage and archiving. To provide near-real-time data download and data analysis service, the data transfer module needs to be designed to be reliable and efficient, while also capable of recording and returning the status of data transmission.

(3) Metadata acquisition and processing – responsible for obtaining metadata from different stages and systems involved in data management and integrating them into the database. How to acquire metadata depends on how it is presented and provided. This module needs to support metadata acquisition from different sources and implement the functions of metadata acquisition, integration and storage through a unified architecture.

(4) Data service – provides users with a web portal for data access, viewing, downloading and offline analysis. Given that this module is typically to be integrated with each facility’s own user service system, the design of the data service needs to fully consider configurability, portability and integrability. The website adopts the MVC design pattern, which separates the data and business logic from the display. Through simple configuration, it can adapt to various data sources and quickly complete the construction of the data service website.

## 3. Overview of DOMAS

According to the design ideas given above, it is clear that DOMAS primarily consists of four functional modules: metadata catalogue, data transfer, metadata acquisition and processing, and data service. By configuring and employing low-code development for these four core modules, we can rapidly develop a data management system tailored to the specific needs of each light source.

**Figure 1:**
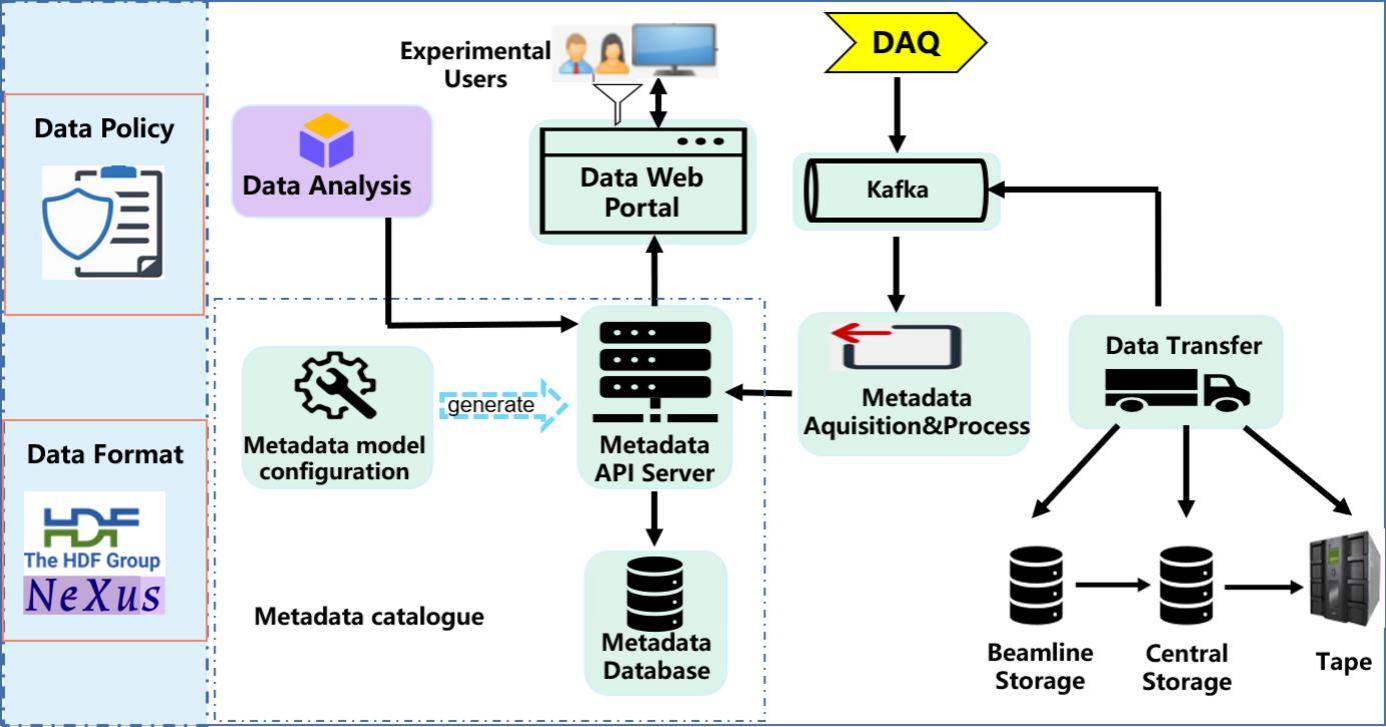
Architecture of DOMAS.

The architecture diagram of DOMAS is shown in Fig. 1 ▸. From the diagram we can see how these four modules communicate and collaborate with each other. The design of the framework follows the scientific data policy of each facility and the data format conventions made by beamlines. The metadata catalogue in the diagram is composed of the metadata API server and the metadata database. The API server provides the metadata access service through RESTful APIs, which are automatically generated by the metadata model configuration through a visual tool. Any metadata access to the API server will automatically be converted into corresponding CURD (create, update, retrieve, delete) operations on the metadata database. Given that metadata comes from multiple sources and has to handle concurrency from multiple beamlines, Kafka message middleware is employed to buffer the metadata. Once the metadata from the DAQ system or data transfer system is sent to the Kafka message queue, the metadata acquisition and processing module consumes metadata from Kafka. On the one hand, it integrates all needed metadata together and saves the metadata to the database. On the other hand, it sends tasks to the transfer module based on the transfer status. This instructs the data transfer module to move data from source storages to destination storages, and relay the transfer status back to the metadata acquisition and processing module via Kafka. At last, experimental users can use the data web portal to search, view, download and analysis their data.

## 4. Functional modules

### 4.1. Metadata catalogue (DOMAS-CAT)

DOMAS-CAT is primarily designed to store the metadata and provide interfaces for accessing this metadata. It utilizes MongoDB as the database for two key reasons. Firstly, it has the capability to store diverse and complicated metadata structures; secondly, it enables high concurrency and performance in reading and writing metadata (Győrödi *et al.*, 2022 ▸). Moreover, DOMAS-CAT offers a visualization tool that generates metadata access interfaces automatically based on metadata model designs.

#### 4.1.1. Functional architecture

**Figure 2:**
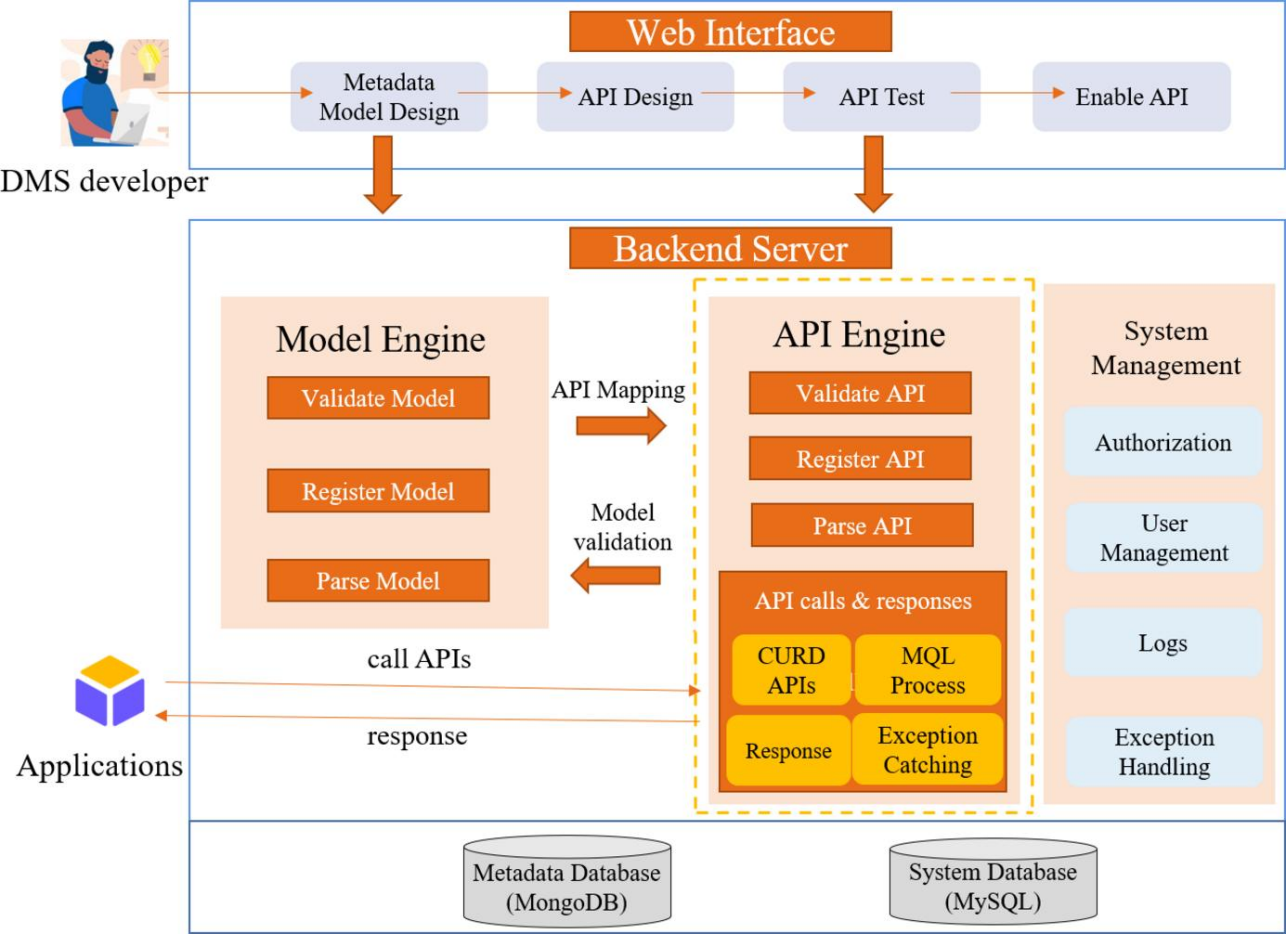
Architecture of DOMAS-CAT.

DOMAS-CAT comprises a web interface and a backend server, as depicted in Fig. 2 ▸. The web interface facilitates metadata model design, API creation, API testing and activation. Data management system (DMS) developers can efficiently generate and publish the necessary APIs through the guided page. The back-end server includes the model engine, API engine and system management. The model engine handles model validation, registration and parsing. The API engine handles API validation, registration, parsing and invocation. System management includes fundamental functions like user and authorization management, exception handling and logging.

Next, let us dive into the interaction flow between the submodules. The entire design is based on the metadata model definition. Firstly, DMS developers design metadata models through the web page. The metadata model, in JSON format, is then sent to the model engine for validation. If the validation adheres to the model specifications, the model is registered in the system database. Next, DMS developers create APIs, and the API, described in JSON format, is sent to the API engine for validation. If it complies with the API specifications, the API is registered. Subsequently, API testing can be performed to ensure correct functionality before activation. Finally, when the APIs are invoked by other applications, the API engine validates the parameters, and converts them into MQL for execution. The entire calling process includes capturing exceptions during execution and returning the results to the API caller.

#### 4.1.2. Metadata model

**Figure 3:**
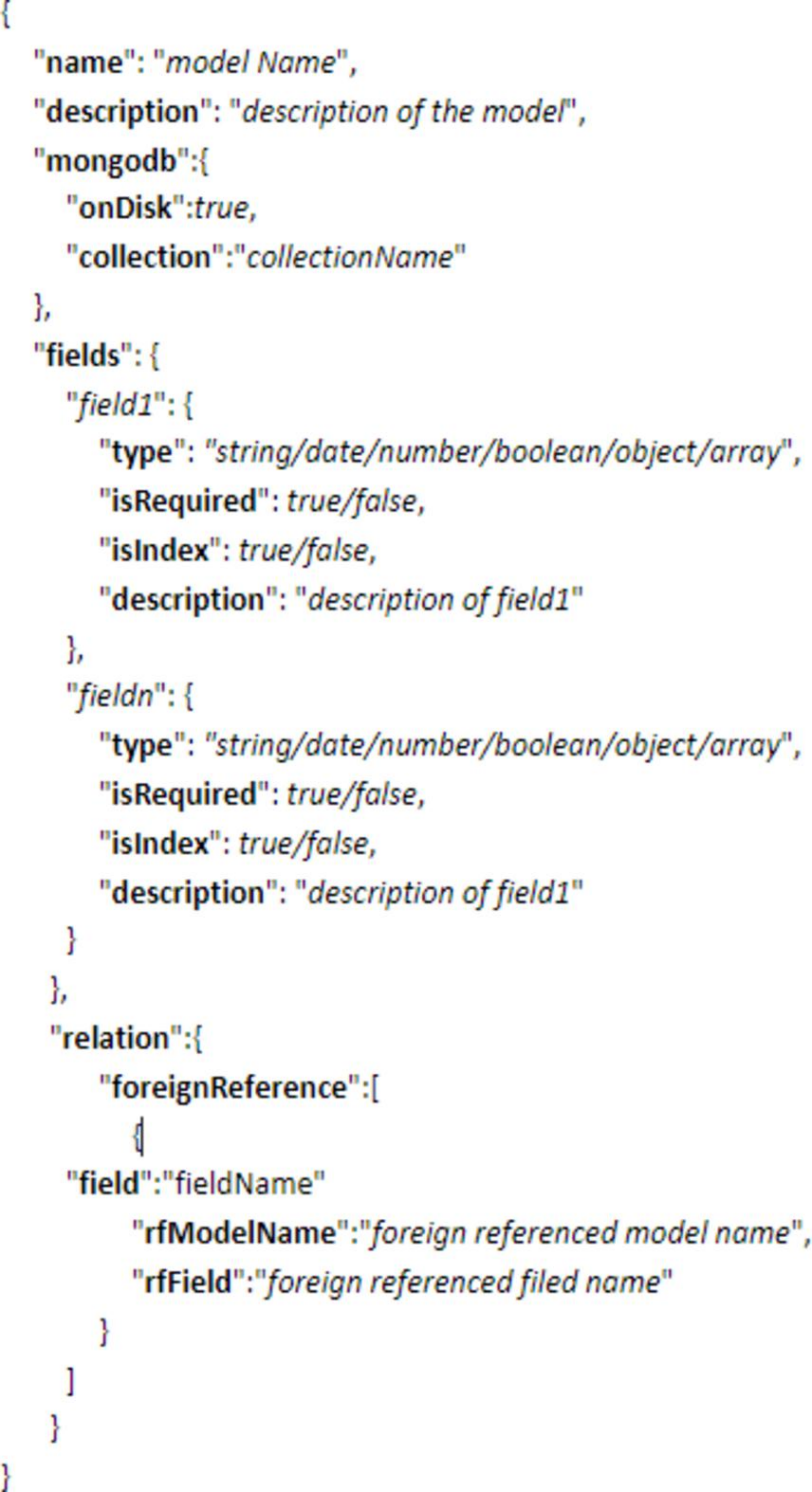
Definition of metadata models.

The metadata model defines the metadata schema using the JSON format, as shown in Fig. 3 ▸. It includes the model name, model description, the collection name in MongoDB and the fields within the collection, as well as the relationships with other models. The field definition specifies the field name, field type, if it is mandatory (Boolean), whether it is indexed, and the description. Currently, only one type of relationship with other models is defined: foreign reference, which can be expanded in the future based on usage requirements.

The metadata model not only leverages the flexibility offered by non-relational databases to store complex structured metadata but also enables the definition of metadata types and relationships between different metadata entities.

#### 4.1.3. The web interface of the metadata model and API design

**Figure 4:**
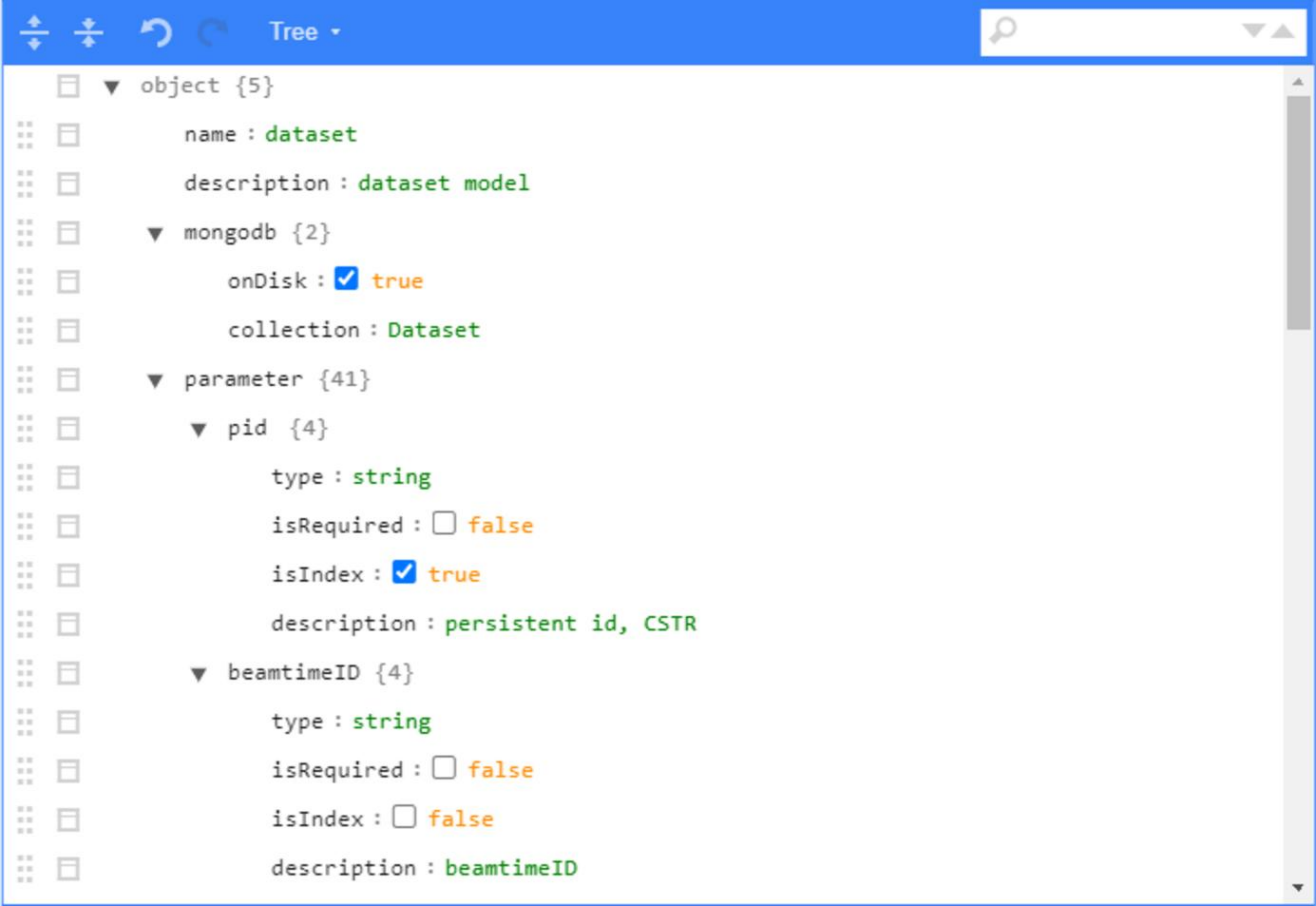
Web interface for model editing with a tree-like structure.

**Figure 6:**
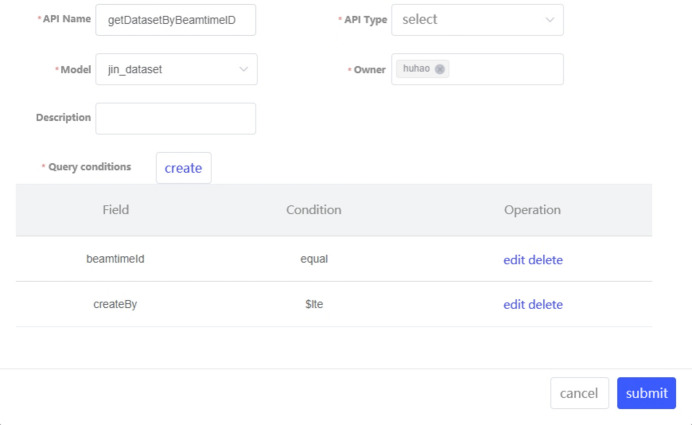
Web interface for API creation.

Figs. 4 ▸ and 5 ▸ display the web interface of the metadata model design. The metadata model can be added and modified using a JSON tree structure diagram. Alternatively, direct JSON editing is also available, and syntax validation is performed before saving the model. On the API creation page, depicted in Fig. 6 ▸, you can choose the API type (add, select, update, delete). For update, select or delete API types, you can set conditions for the API operation. Different data types will correspond to different query conditions.

### 4.2. Metadata acquisition and processing

**Figure 7:**
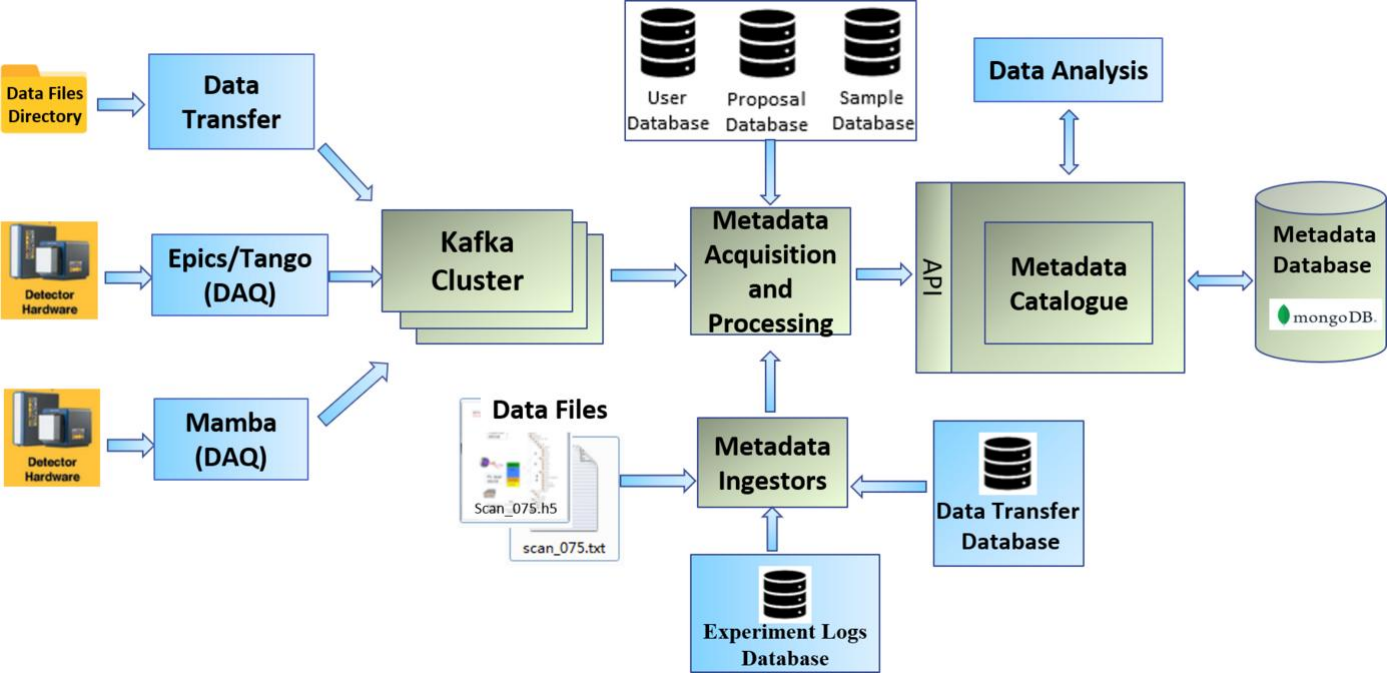
Architecture of metadata acquisition and processing.

The metadata to be acquired for advanced light sources include administrative metadata and scientific metadata. These metadata need to be extracted from various subsystems involved in the entire lifecycle processes of data acquisition, data storage, data transfer, data analysis and data publication. Therefore, the challenge of metadata acquisition is the diversity of its sources, and the acquisition methods are closely related to the ways metadata are provided in each subsystem. Consequently, we designed an architecture for multi-source metadata acquisition, illustrated in Fig. 7 ▸. This structure employs various metadata acquisition plugins to extract metadata from different interfaces, which are then sent to the Kafka brokers. Subsequently, the extracted metadata is associated and integrated and then saved to the metadata database through the API provided by the metadata catalogue.

#### 4.2.1. Metadata acquisition

As we know, metadata may come from various sources such as the DAQ systems of different detectors, data transfer modules, data files or experiment logs, and this architecture allows metadata acquisition to be loosely coupled between subsystems. Generally, we can receive metadata through Kafka as the messaging interface. However, if that is not available, metadata can be obtained by developing metadata ingestors. These metadata ingestors will run flexibly as plugins (Schwarz *et al.*, 2019 ▸). Currently, two types of metadata ingestors are provided:

(i) File-based ingestors – extracting metadata from files in TXT, HDF5 (HDF Group, 2018 ▸) or NXS format, when the detector is a commercial product without related interfaces and the metadata are collected and saved to these files.

(ii) Database ingestors – directly acquiring metadata from databases of other subsystems, such as DAQ, data transfer or E-logbook

#### 4.2.2. Metadata processing

The metadata processing functionality aims to collect and integrate metadata from different data sources. For example, it can associate and integrate user information, proposal information and sample-related information from databases such as user database, proposal database or sample database. This ensures the correct association between various metadata.

The specific approach to integrate and associate metadata will vary depending on how each facility or beamline station designs their metadata storage. Therefore, this part of the work needs more development work according to the data management system design of the facility.

### 4.3. Data transfer

Data transfer is an essential part of the data management software framework for advanced light sources. It is responsible for implementing near-real-time, efficient, reliable and fully automated migration of experimental data between different storage devices.

In order to balance the cost-effectiveness of storage devices and realize the high reliability of data storage, advanced light sources often employ hierarchical storage and long-term archive for experimental data. Taking HEPS as an example, a three-tier storage is designed, including beamline storage, central storage and tape (Cano *et al.*, 2020 ▸). To analyse and download data in near-real-time, the data produced from detectors at beamlines are first saved to the beamline storage. Subsequently, the data transfer module moves the data automatically from the beamline storage to the central storage, and then to the tape. Additionally, data will be automatically and efficiently moved from tape storage to central storage based on the user’s access and analysis requirements.

#### 4.3.1. The functional and deployment architecture

**Figure 8:**
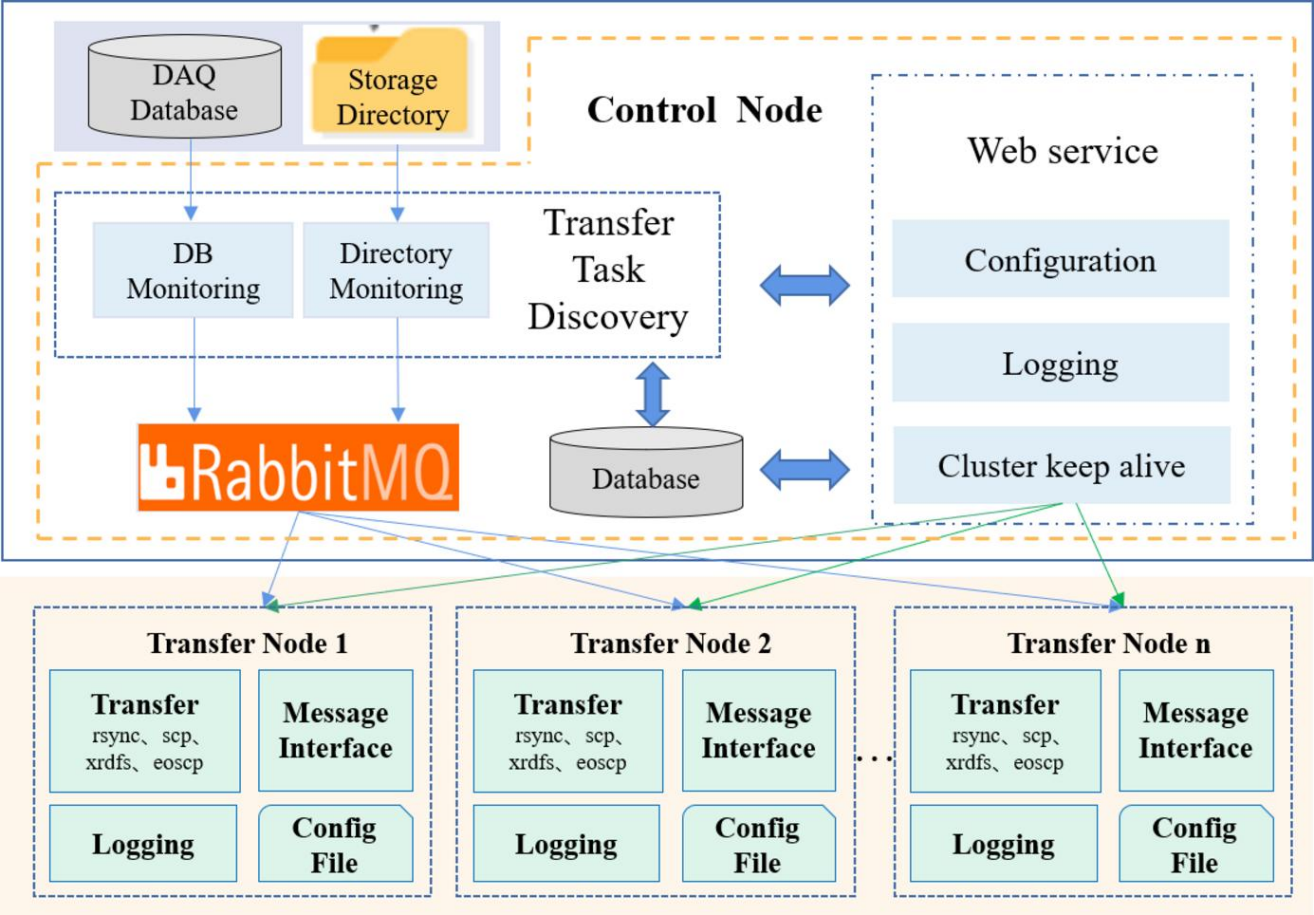
Functional and deployment architecture of the data transfer cluster.

The data transfer supports the cluster mode to achieve high concurrency and scalability of the system, consisting of a control node and one or multiple transfer nodes. The functional and deployment architecture of the data transfer cluster is shown in Fig. 8 ▸.

The controller node contains plugins for transfer task discovery, message queue (RabbitMQ), and provides web services for administrators to configure transfer tasks, view transfer status and logs, and manage cluster status. The transfer node is responsible for transferring files using different protocols, logging the transmission process, interacting with other systems through message interfaces, and keeping their own configuration files.

Before the startup of data transfer, some related parameters, such as plugins for transfer task discovery, transfer protocols, transfer processes numbers and triggering events, need to be configured through the web service of the control node.

After data transfer is started, the transfer task discovery can detect the files that need to be transferred and create a file list, which is then sent to the RabbitMQ. The transfer nodes consume the transfer tasks from the RabbitMQ and start to use the specified transfer protocol or command to transfer the data from the source path to the destination path.

The logging module records the detailed transmission process in files and writes the status of success or failure to the database in the control node. Additionally, the transfer nodes are able to send messages using the configured message interface after the transfer, such as RabbitMQ or Kafka, to interact with other systems.

#### 4.3.2. Transfer task discovery

Two modes of discovering transfer task are supported: directory monitoring and database polling.

When the data acquisition system stores metadata of the generated data in a DAQ database, the transfer tasks are detected by periodically polling the DAQ database. Generally, the DAQ database contains records of the generated experimental data, including the file name, the file path, and the creation time and the checksum of data files. All the metadata are sent to the RabbitMQ for subsequent processes.

When there is no DAQ database in the data acquisition system, the transfer tasks can only be obtained through directory monitoring. However, directory monitoring faces two difficulties. First, it is difficult to accurately determine whether a file has been completely written and its handle has been closed before it is transferred. Second, there are a large number of directories to monitor which can result in high resource consumption and low efficiency in monitoring. Both challenges have been improved through the following optimizations:

(i) To ensure the integrity of files, it is good practice to compare the file size and modification time (mtime) within a reasonable time period to determine whether the file has been fully written. The optional time period setting may vary depending on different data writing modes of each beamline.

(ii) The monitoring directories can be customized by configuring multi-level directory matching rules and file type settings. Regular expressions are used to define the scanning path range. Moreover, the system can limit the monitoring scope to recently created directories based on their creation time, thereby further reducing the number of directories to monitor.

#### 4.3.3. Transfer

The transfer submodule starts the transfer process based on the configuration and utilizes the corresponding transfer protocol. Multiple transfer protocols are integrated as plugins, including rsync, scp, xrdcp and eoscp. For light source facilities, data synchronization or transfer typically takes place within a highly reliable and high-bandwidth intranet environment; there is no performance issue with the transfer protocols. However, if there is a requirement to transfer data over a wide area network with limited bandwidth guarantees, it is possible to integrate other open-source, commercial or self-developed transfer tools to further improve the efficiency of data transfer.

In terms of reliability, the transfer submodule performs data verification and initiates file retransmission in case of failures. The file integrity is verified by performing checksum validation. If a transfer fails, the file will be placed back into the transfer message queue for retransmission. If a file fails to transfer more than five times, it is marked as an exceptional file for manual retransmission or further handling.

#### 4.3.4. Logging and monitoring

**Figure 9:**
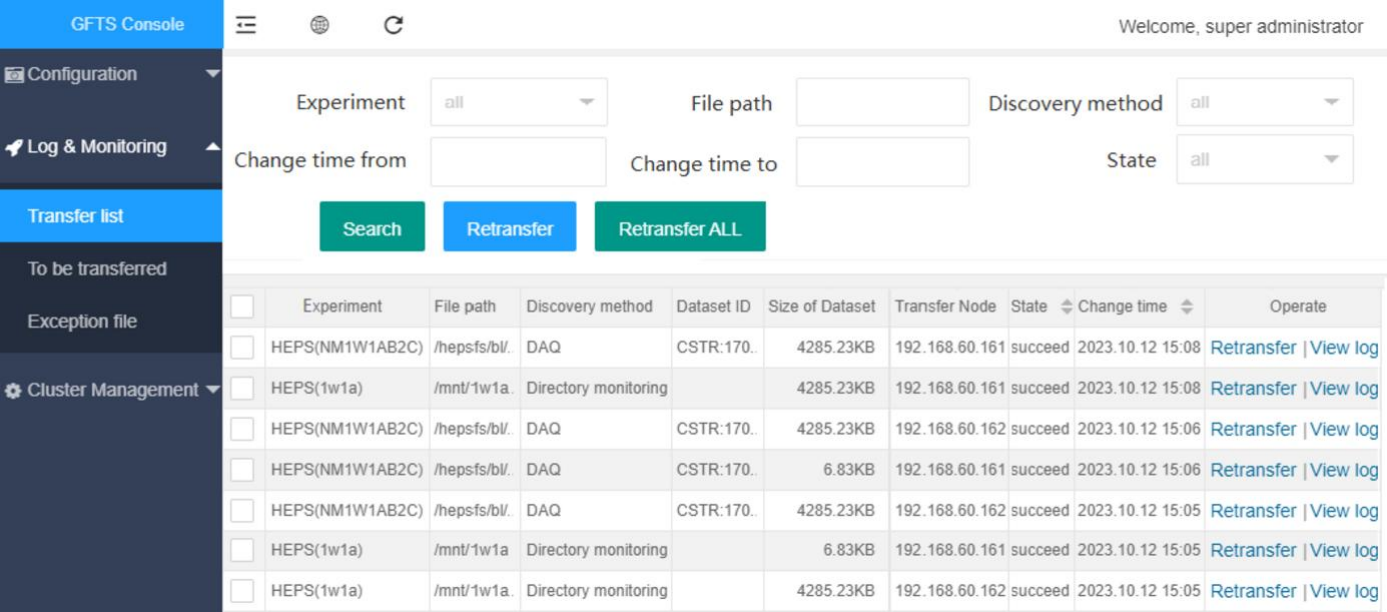
Web interface for transfer logs.

One crucial functionality of data transfer is logging and monitoring (see Fig. 9 ▸). It records the detailed process of file transfers, including the file name, start and end time of the transfer, file size, checksum, source and destination path. It is particularly important to log information related to transfer failures and exceptions, such as failed file name, the reason for the exception, and the number of failed attempts. These log details can be queried and displayed through a web page, including the task list, transfer status, and file lists with transfer exceptions. It helps us to stay informed about the status of data transfers at any given time and enables us to swiftly troubleshoot and diagnose issues and failures.

### 4.4. Data service

Data service primarily focuses on delivering a comprehensive set of features to users through a data web portal (Birkle *et al.*, 2020 ▸). These features include essential functions like data retrieval, preview and download, as well as other services such as data authorization, analysis and the ability to view experimental logs. By utilizing a data web portal, users can efficiently access, explore and manipulate data while ensuring data security, facilitating analysis and promoting transparency in research activities (Corti *et al.*, 2019 ▸).

The data service offers a website with fundamental functionalities, designed to deploy independently or seamlessly integrate with user service system of each light source facility. This website not only allows for flexible feature configuration but also provides a solid foundation for secondary development and customization.

**Figure 10:**
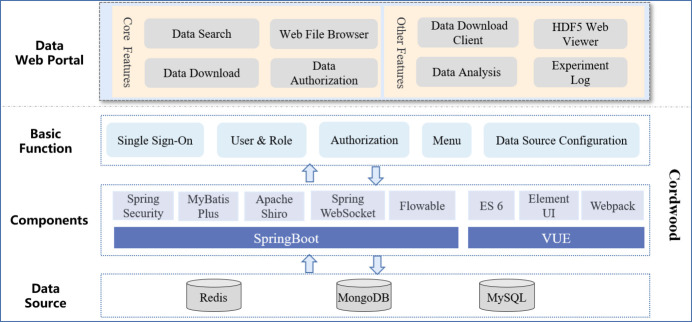
Architecture of data service.

From Fig. 10 ▸, we can see the development architecture of the data service. The data service is built base on Cordwood, a low-code website development platform developed by IHEP and soon to be open-sourced. Cordwood uses a front-end and back-end separation structure and a micro-service architecture. The back-end is developed in JAVA and is based on the SpringBoot2.5 framework (Suryotrisongko *et al.*, 2017 ▸), including Spring Security and Apache Shiro for security, Mybatis-Plus for data persistence, Redis for data caching, and other components. The front-end utilizes technologies such as VUE 2.0 and Element UI. Cordwood provides basic functionalities for website building, including single sign-on authentication, user roles and authorization, menu customization and data source configuration.

**Figure 11:**
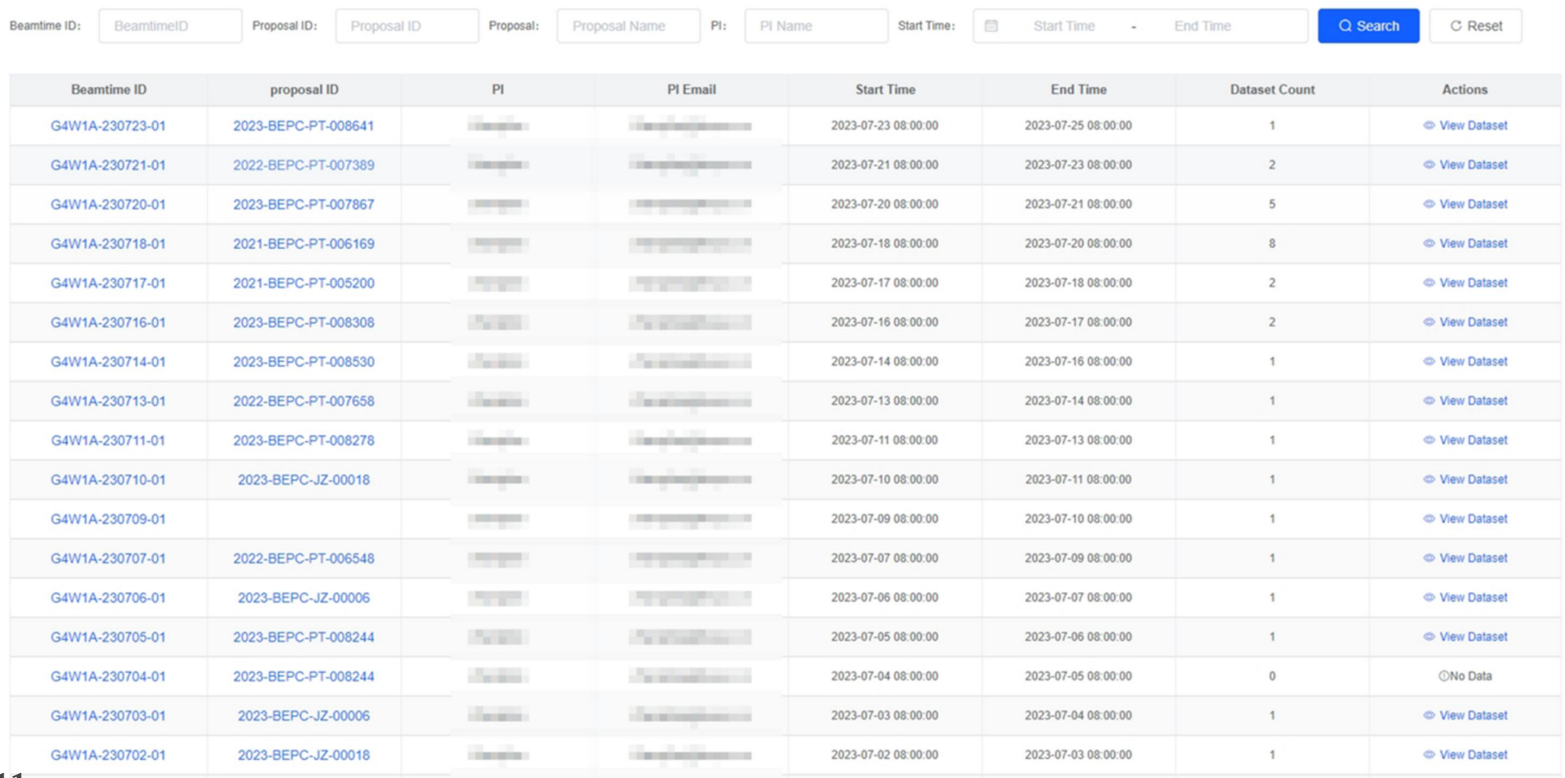
Web interface for dataset searching through metadata retrieval.

The core features of a data web portal include data search, data download, web file browser and data authorization. Experimental users can search for data by metadata retrieval or browse data through a tree directory structure, as shown in Figs. 11 ▸ and 12 ▸.

**Figure 13:**
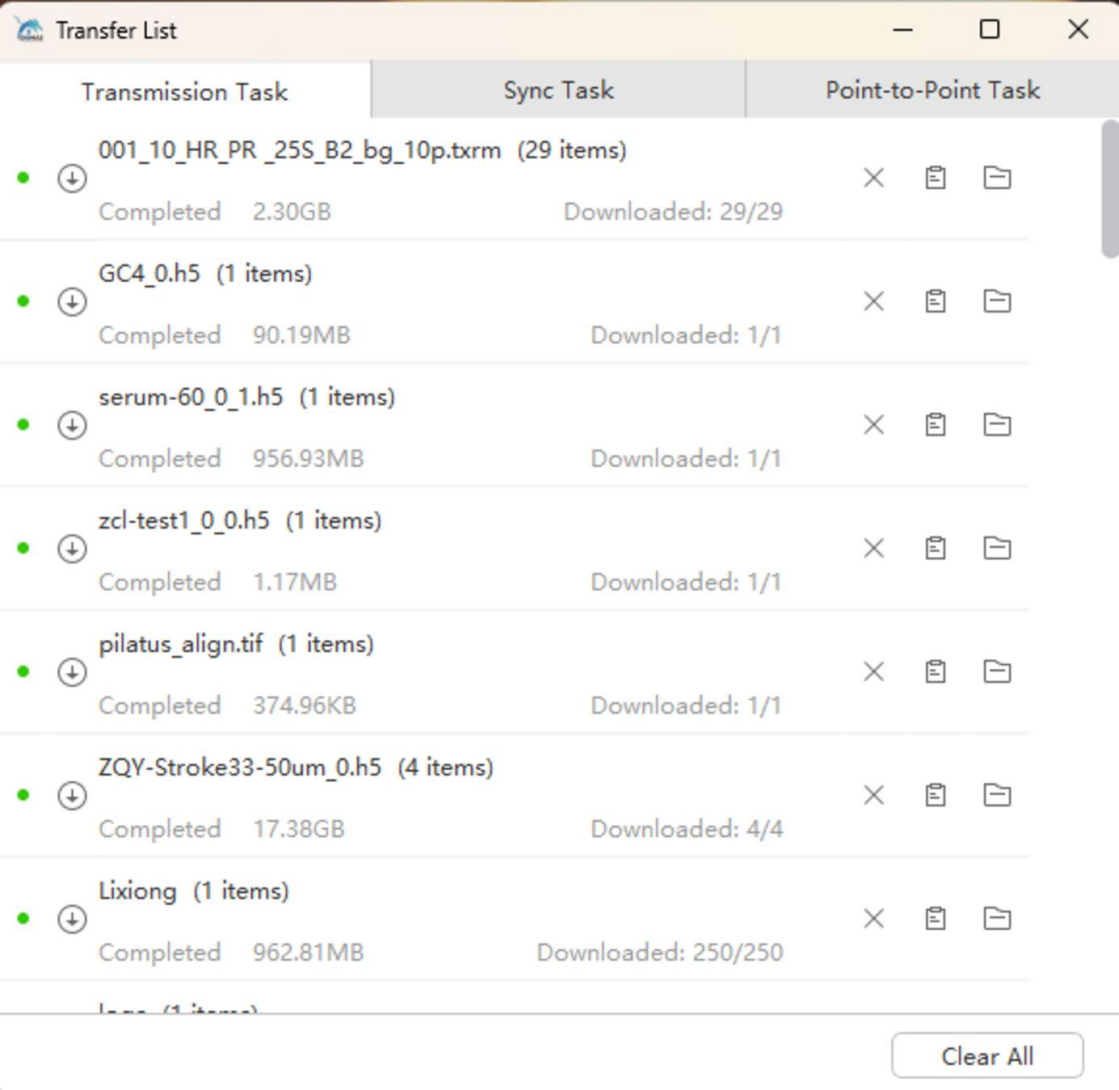
Web page showing the download progress of files.

Given that data download is the most commonly used function on the data web portal, both HTTP download and client-based download options will be provided. However, the download speed is often limited by the user’s local network bandwidth. When dealing with large data volumes, the speed of HTTP download is unsatisfactory and unacceptable. In such cases, high-speed client-based download is recommended. Once all the desired files are selected for download, clicking the ‘Batch High-Speed Download’ button will launch the client for download. Users can view the file list, download progress and download speed, and these web pages are shown in Figs. 13 ▸, 14 ▸ and 15 ▸. The integrated high-speed download client can perform parallel transmission of multiple files and incorporates lossless compression algorithms, enabling maximum bandwidth utilization and supporting resumable downloads. We tested the download of four 4.49 GB files in a 1000M network environment – it took 2 minutes and 28 s, achieving an average download speed of 839 Mb s^−1^. This download speed is already very satisfactory. However, if you want to download extremely large data from the data web portal, such as tens or hundreds of terabytes, the time it takes can be intolerable. In this case it is necessary to consider providing a dedicated data download terminal on the facility for users to copy the data.

In addition, a data web portal can be enhanced with extensible features by links. For example, (i) HDF5 web viewer, allowing users to preview HDF5 files on the web; (ii) an entry point of data analysis that launches the required computing resources and environment, enabling users to perform data analysis in a virtual cloud desktop or in JupyterLab; (iii) links to experimental logs by integrating with an e-log system, allowing users to view relevant logs when accessing experimental data.

## 5. Progress and applications

The core functional module of DOMAS has been completed, and we are progressively open-sourcing each module. DOMAS has been applied to HEPS. Based on DOMAS, combined with the specific scientific data policy of HEPS, standardized data file formats and data storage directory designs, we have completed the development of the scientific data management system for HEPS. At the same time, the system also implemented the fully automated movement and management of data among three levels of storage (beamline storage, central storage and tape). To ensure that the HEPS data management system can operate normally and stably when HEPS enters the trial operation phase in 2024, the HEPS data management system has undergone functional and process verification at multiple beamlines of Beijing Synchrotron Radiation Facility (BSRF), and is stably running in the formal environment of the X-ray fluorescence microanalysis beamline station, providing users with data retrieval, viewing, downloading and analysis services.

In the process of applying DOMAS to HEPS, we found that DOMAS still needs further optimization. DOMAS-CAT is not flexible enough in interface generation. In the future, we hope to design interfaces that include multiple CURD combinations through the interface, to realize more complex process logic. The data transfer module can further improve transmission efficiency by optimizing the transfer protocol, and can flexibly embed some algorithms for data compression or data format conversion, in order to reduce the storage space when archiving massive amounts of data.

## 6. Conclusion and plan

In response to the common needs and challenges of scientific data management for advanced light sources in China, we have encapsulated metadata catalogue, metadata acquisition and processing, data transfer and data service into common modules, providing general interfaces to form the scientific data management software framework DOMAS. The application of DOMAS allows advanced light sources to quickly develop and deploy data management systems, enabling efficient management, tracking and utilization of experimental data, while saving time and manpower investment in scientific data management development for each facility.

DOMAS has already finished the functionality and process verification at BSRF and HEPS, making significant progress. Meanwhile, the data teams at SHINE and HALF have decided to apply DOMAS to the development of their respective scientific data management systems. Our future plans involve enhancing and broadening the capabilities of the framework, such as the integration of a workflow module. We also hope to extend the application of DOMAS to a wider range of large scientific facilities, such as the China Spallation Neutron Source (CSNS) and the High Energy cosmic-Radiation Detection facility (HERD). This is with the goal of establishing DOMAS as a universal solution for data management across large scientific facilities domestically.

## Figures and Tables

**Figure 5:**
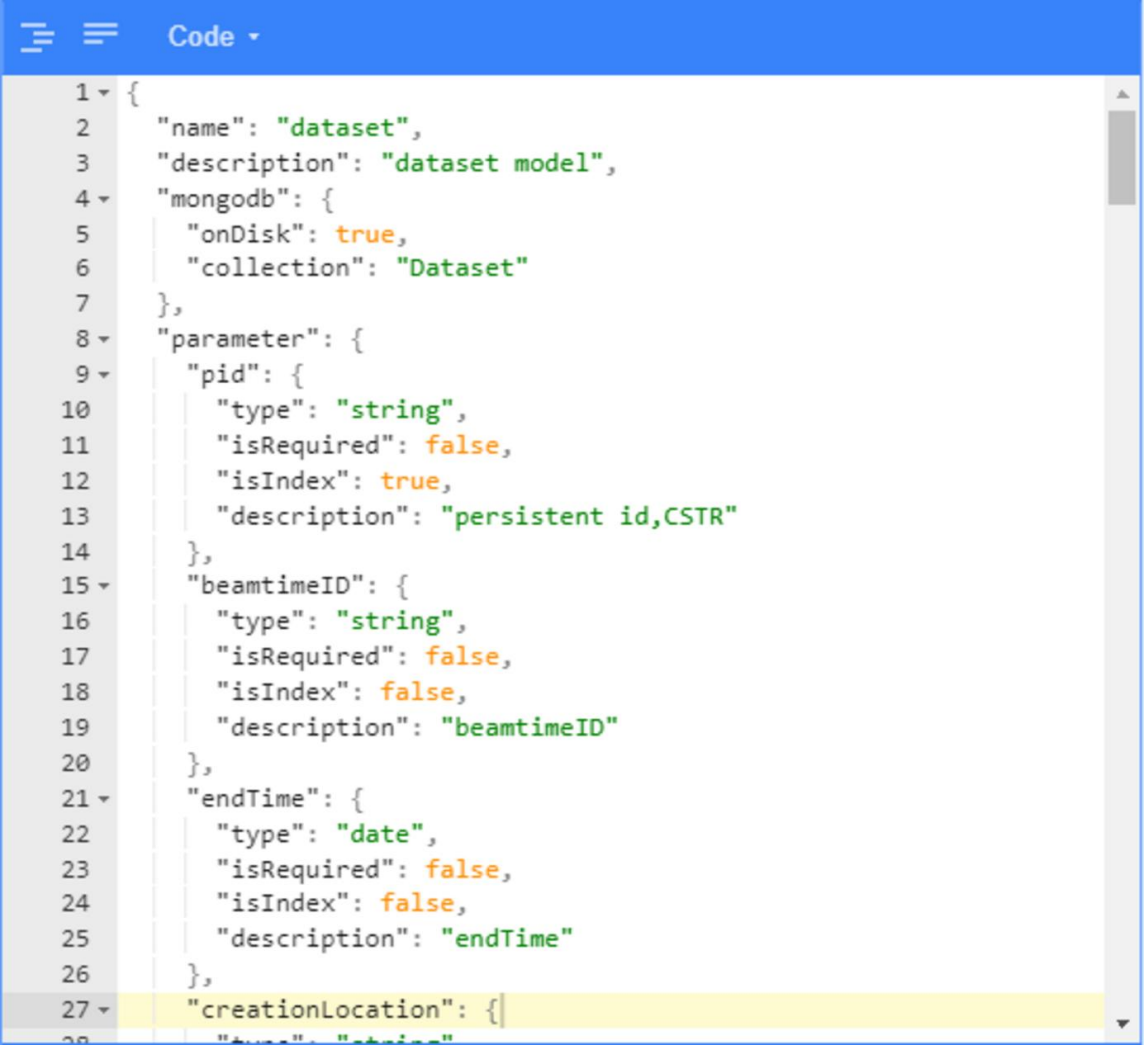
Web interface for model editing with JSON format.

**Figure 12:**
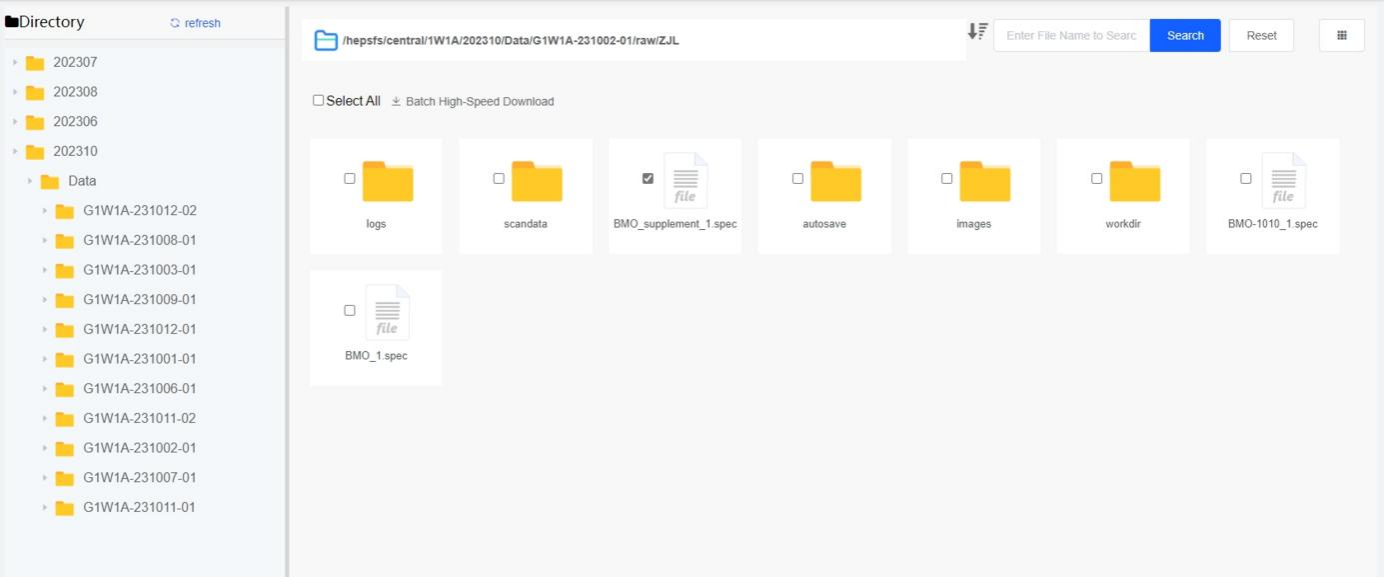
Web interface for file browser.

**Figure 14:**
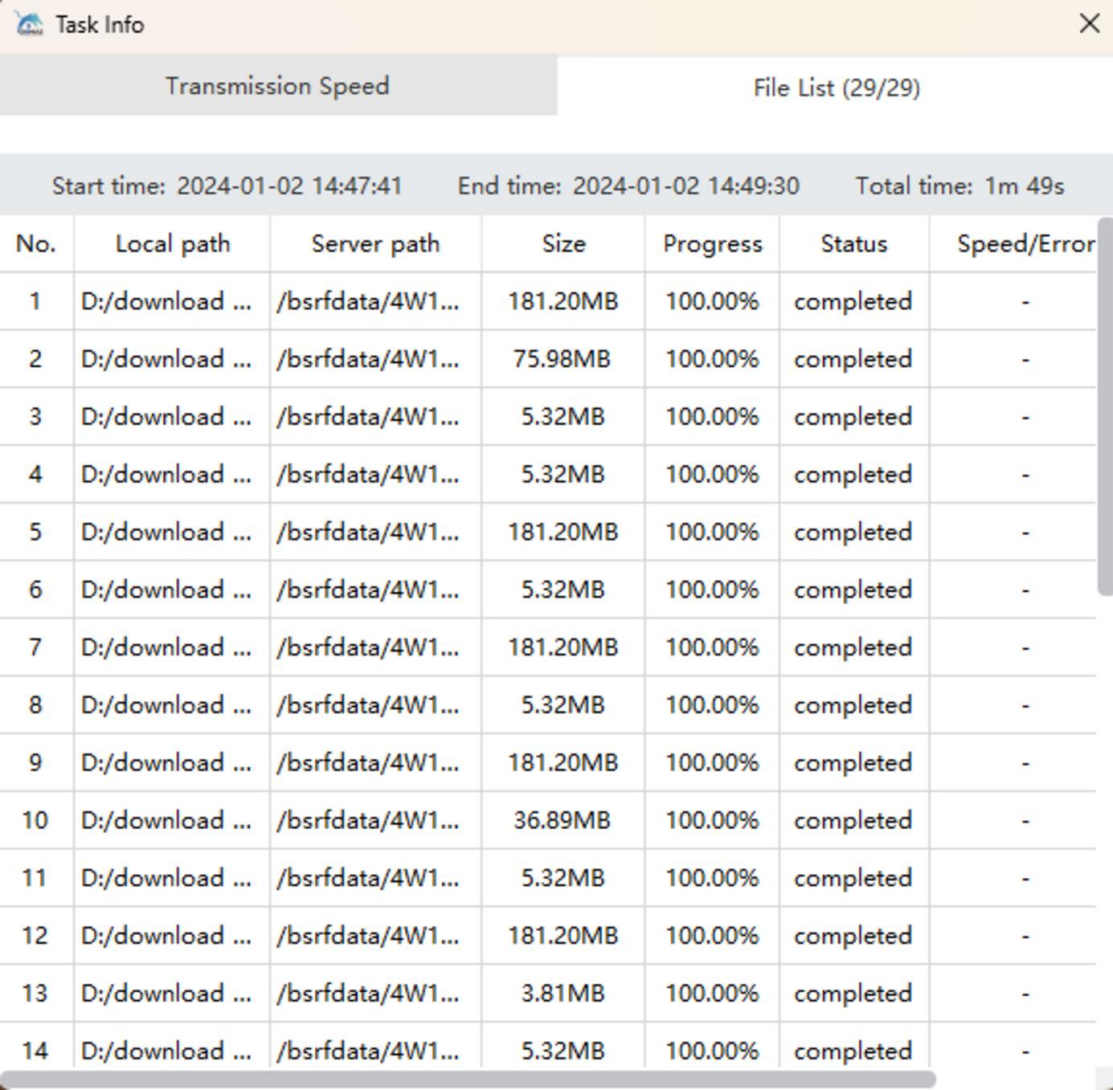
Web page showing the list of downloaded files.

**Figure 15:**
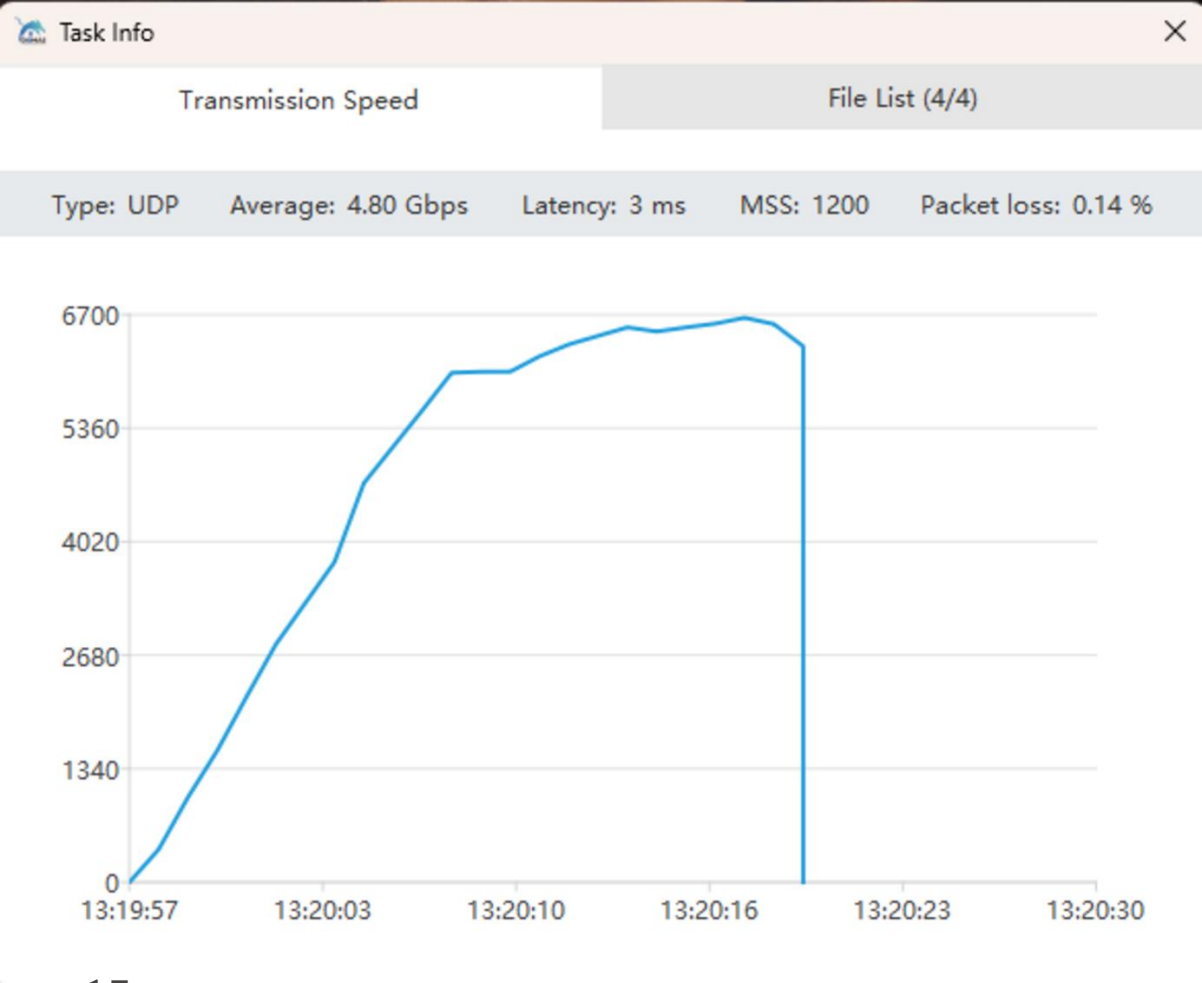
Web page showing the download speed of files.
